# Causal associations between telomere length and pulmonary arterial hypertension: A two-sample Mendelian randomization study

**DOI:** 10.1097/MD.0000000000040407

**Published:** 2024-11-22

**Authors:** Ting-Ting Lyu, Jing-Yang Wang, Jiang-Shan Tan, Yan-Min Yang, Yi-Meng Wang, Jing Zhao, Ping Qing, Ling-Min Wu, Xiao-Jian Wang

**Affiliations:** a State Key Laboratory of Cardiovascular Disease, Fuwai Hospital, National Center for Cardiovascular Diseases, National Clinical Research Center of Cardiovascular Diseases, Chinese Academy of Medical Sciences, Peking Union Medical College, Beijing, China; b Emergency and Critical Care Center, Fuwai Hospital, National Center for Cardiovascular Diseases of China, Chinese Academy of Medical Sciences and Peking Union Medical College, Beijing, China; c National Health Commission Key Laboratory of Cardiovascular Regenerative Medicine, Fuwai Central-China Hospital, Central-China Branch of National Center for Cardiovascular Diseases, Zhengzhou, China; d Key Laboratory of Pulmonary Vascular Medicine, Fuwai Hospital, National Center for Cardiovascular Diseases, Chinese Academy of Medical Sciences and Peking Union Medical College, Beijing, China; e Fuwai Yunnan Cardiovascular Hospital, Kunming, China.

**Keywords:** causal association, Mendelian randomization, pulmonary arterial hypertension, telomere length, two-sample

## Abstract

Pulmonary arterial hypertension (PAH) is a life-threatening condition characterized by elevated pulmonary artery pressure, leading to right heart failure, and mortality. The role of telomere length, a marker of biological aging, in PAH remains unclear. We utilized summary-level data from genome-wide association studies for various measures of telomere length and PAH. Single nucleotide polymorphisms associated with telomere length at a genome-wide significance level were used as instrumental variables. The inverse variance weighted method was the primary analysis, with sensitivity analyses including the weighted median and Mendelian randomization-Egger regression. The odds ratios and 95% confidence intervals (CI) were calculated to estimate the causal effect of telomere length on PAH risk. The Mendelian randomization analyses revealed no significant causal association between overall telomere length and PAH (odds ratios per standard deviation increase = 1.229, 95% CI: 0.469–3.222, *P* = .676). Similar null findings were observed for granulocyte, lymphocyte, naive T-cell, memory T-cell, B-cell, and natural killer-cell telomere lengths. Sensitivity analyses confirmed the robustness of the results, with no evidence of horizontal pleiotropy or significant influence of individual single nucleotide polymorphisms on the overall estimates. This Mendelian randomization study didn’t support a causal association between telomere length and PAH.

## 1. Introduction

Pulmonary arterial hypertension (PAH) is a progressive disorder characterized by elevated blood pressure in the pulmonary arteries, leading to right ventricular failure and, ultimately, death if untreated.^[1]^ Despite its low prevalence, PAH is associated with significant morbidity and mortality, highlighting the need for a deeper understanding of its pathogenesis and risk factors.^[2]^ Genetic predisposition plays a critical role in the etiology of PAH, particularly in familial PAH . It is estimated that mutations occur in approximately 70% of patients with familial PAH, highlighting the significant genetic component underlying this condition.^[3]^ In addition, a substantial proportion of idiopathic PAH cases, which lack a clear familial link, also exhibit genetic mutations, with approximately 20% of adults and 36% of children with idiopathic PAH harboring such mutations.^[4]^ These findings underscore the importance of genetic factors in the development of PAH. Several genetic association studies have identified risk alleles in key genes involved in endothelial function and vascular remodeling.^[4]^ However, the genetic architecture of PAH is complex, and the contribution of individual genetic variants to disease risk is not fully understood.

Telomeres, the protective caps at the ends of chromosomes, have emerged as critical players in cellular aging and senescence.^[5]^ The shortening of telomeres with each cell division is a hallmark of biological aging and is associated with an increased risk of age-related diseases, including cardiovascular diseases.^[6]^ In the context of pulmonary disease, shortened telomeres have been linked to idiopathic pulmonary fibrosis and chronic obstructive pulmonary disease, suggesting a potential role in lung pathophysiology.^[7–9]^ Most importantly, idiopathic PAH is more common in elderly patients (>65 years) when compared with younger (18–65 years),^[10]^ suggesting that PAH may be associated with age.

Despite preliminary evidence suggesting an association between telomere length and PAH,^[11]^ the nature of this relationship remains unclear. Observational studies have been limited by confounding factors and the inability to establish causality.^[12]^ To overcome these limitations, Mendelian randomization (MR), which uses genetic variants as instrumental variables (IVs) to infer causal relationships between modifiable exposures and disease outcomes, offers a promising approach.^[13]^ MR is an increasingly popular analytical method in cardiovascular research for investigating the causal effects of modifiable risk factors on cardiovascular outcomes.^[13–15]^ By leveraging genetic variants associated with telomere length, MR can provide insights into the causal role of telomere biology in the development of PAH.

Given the gap in current knowledge, this study aims to determine the causal association between telomere length and PAH using a two-sample MR approach. Understanding the contribution of telomere biology to PAH could shed light on disease mechanisms and potentially identify novel therapeutic targets for this debilitating condition.

## 2. Methods

### 2.1. Study design

**Figure 1.**
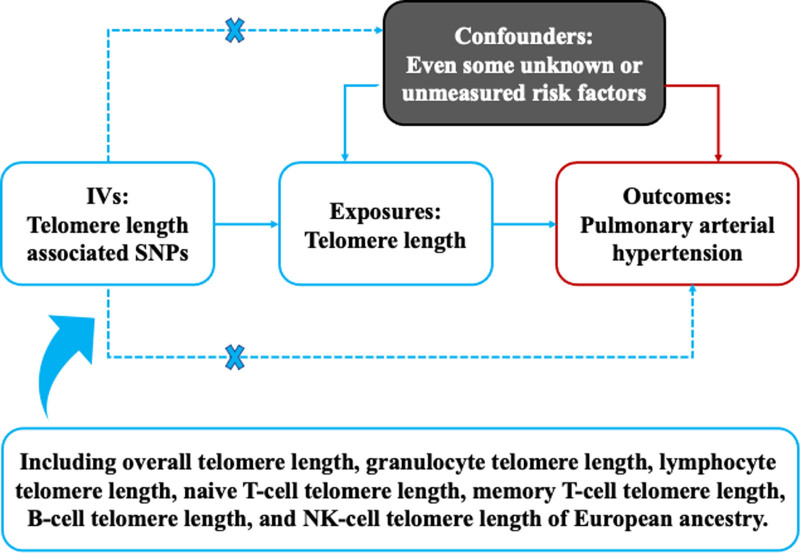
The design of the present Mendelian randomization study. This figure illustrates a Mendelian randomization framework used to investigate the causal relationship between telomere length and pulmonary arterial hypertension. Arrows depict the hypothesized causal pathways, with genetic instruments estimating the effect of telomere length on pulmonary arterial hypertension. Crosses on confounder pathways indicate the exclusion of these variables from the causal pathway in the Mendelian randomization analysis, enhancing the validity of the causal inferences. IVs = instrumental variables; NK = natural killer; SNPs = single nucleotide polymorphisms.

This study employed a two-sample MR approach to investigate the causal association between telomere length and PAH (Fig. 1). Utilizing genetic variants as IVs, the MR framework mimics the principles of a randomized controlled trial, ensuring that the IVs are randomly allocated during gametogenesis.^[14]^ This design enables the inference of causal associations between the exposure (including telomere length, granulocyte telomere length, memory T-cell telomere length, naive T-cell telomere length, lymphocyte telomere length, B-cell telomere length, natural killer (NK)-cell telomere length) and the outcome (PAH) based on summary-level data from genome-wide association studies (GWASs).^[15]^ Ethical approvals and informed consent procedures for the original studies included in this analysis were adhered to, negating the need for additional ethical clearance for this MR study.^[12]^

This figure illustrates a MR framework used to investigate the causal relationship between telomere length and PAH. Arrows depict the hypothesized causal pathways, with genetic instruments estimating the effect of telomere length on PAH. Crosses on confounder pathways indicate the exclusion of these variables from the causal pathway in the MR analysis, enhancing the validity of the causal inferences.

### 2.2. Data sources

The exposure variable of interest, telomere length, was characterized by genetic variants derived from the most comprehensive and contemporary GWAS specifically focused on telomere length.^[16]^ This GWAS encompassed a variety of telomere length measurements,^[16]^ including overall telomere length, granulocyte telomere length, memory T-cell telomere length, naive T-cell telomere length, lymphocyte telomere length, B-cell telomere length, and NK cell telomere length. Detailed information about the included studies is shown in Table 1.

**Table 1:** Information on used studies and consortia.

Exposure or outcome	Unit	Participants	Identified SNPs	All SNPs
Telomere length*	SD	472,174	224	20,134,421
Granulocyte telomere length^[16]^	SD	902	7	5,213,200
Lymphocyte telomere length^[16]^	SD	902	6	5,213,200
Naive T-cell telomere length^[16]^	SD	902	4	5,213,200
Memory T-cell telomere length^[16]^	SD	902	6	5,213,200
B-cell telomere length^[16]^	SD	902	5	5,213,200
Natural killer -cell telomere length^[16]^	SD	902	5	5,213,200

*Note*: All of the studies were finished in the European populations and built on the HG19/GRCh37.

SD = Standard deviation, SNPs = single nucleotide polymorphisms.

* The SNPs were extracted from the GWAS pipeline using Phesant-derived variables from UKBiobank.

For the outcome, PAH, summary-level data were obtained from the largest available GWAS on PAH, which included 125 cases and 218,667 controls of European descent. This ensured the robustness and representativeness of the outcome data for the analysis. The above data were obtained from publicly available genome-wide association study data, and no additional ethical approval was required.

### 2.3. Instrument variable selection

The selection of IVs for this MR study was a critical step in ensuring the robustness and validity of the causal inference. The IVs were single nucleotide polymorphisms (SNPs) associated with exposures. To meet the 3 basic assumptions of MR, the following criteria were applied in the selection of IVs^[12]^: (1) Relevance: The IVs must be significantly associated with the exposure of interest, telomere length, at a genome-wide significance level (*P* < 5 × 10^−6^), which was chosen based on its use in several recent MR studies and have demonstrated its adequacy for identifying robust instruments while maintaining sufficient statistical power.^[17,18]^ (2) Independence: The IVs should not be associated with any confounders that could influence both the exposure and the outcome. To ensure independence, linkage disequilibrium pruning was performed using an *r*^2^ threshold of 0.001 and a clumping window of 10,000 kilobases, which ensured that the selected SNPs were independent of each other and not in linkage disequilibrium with other genetic variants that could act as confounders. (3) Exclusion-Restriction: The IVs should influence the outcome (PAH) only through their effect on the exposure (telomere length). This assumption is inherently challenging to test directly, but several strategies were employed to minimize the risk of violation, which included selecting SNPs specifically associated with telomere length rather than other traits and conducting sensitivity analyses to detect and account for potential pleiotropy.^[19]^

The selection process involved extracting SNPs from the GWAS summary statistics using the “extract_instruments” function, followed by linkage disequilibrium-based clumping to ensure that only independent and significantly associated SNPs were included as IVs. The final set of SNPs used as IVs in this MR analysis underwent rigorous filtering to meet the aforementioned criteria, thereby enhancing the credibility and validity of the IVs for this study.

### 2.4. Statistical analysis

The primary analysis was conducted using the inverse variance weighted (IVW) method under the assumption of valid IVs.^[12]^ To ensure the robustness of our findings, sensitivity analyses were carried out employing the weighted median approach and MR-Egger regression to account for potential pleiotropy.^[15]^ The presence of horizontal pleiotropy was evaluated using the MR-Egger intercept test, while heterogeneity among the IVs was assessed via Cochran's Q test.

To quantify the causal relationship between telomere length and pulmonary hypertension, odds ratios (OR) and 95% confidence intervals (CI) were calculated, with statistical significance set at *P* < .05. The analyses were performed using the TwoSampleMR package (version 0.5.6) for the MR-Egger and weighted median methods. The consistency of results across the different analytical methods bolstered confidence in the stability and reliability of the causal inferences drawn in this MR study.

## 3. Results

### 3.1. Genetic IVs for exposures

**Figure 2.**
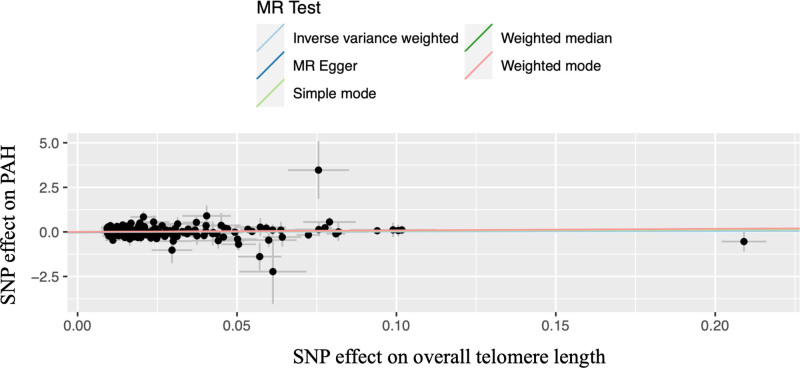
The scatter plot for the causations between telomere length and the risk of PAH. MR: Mendelian randomization; PAH: pulmonary arterial hypertension; SNP, single nucleotide polymorphism.

**Figure 3.**
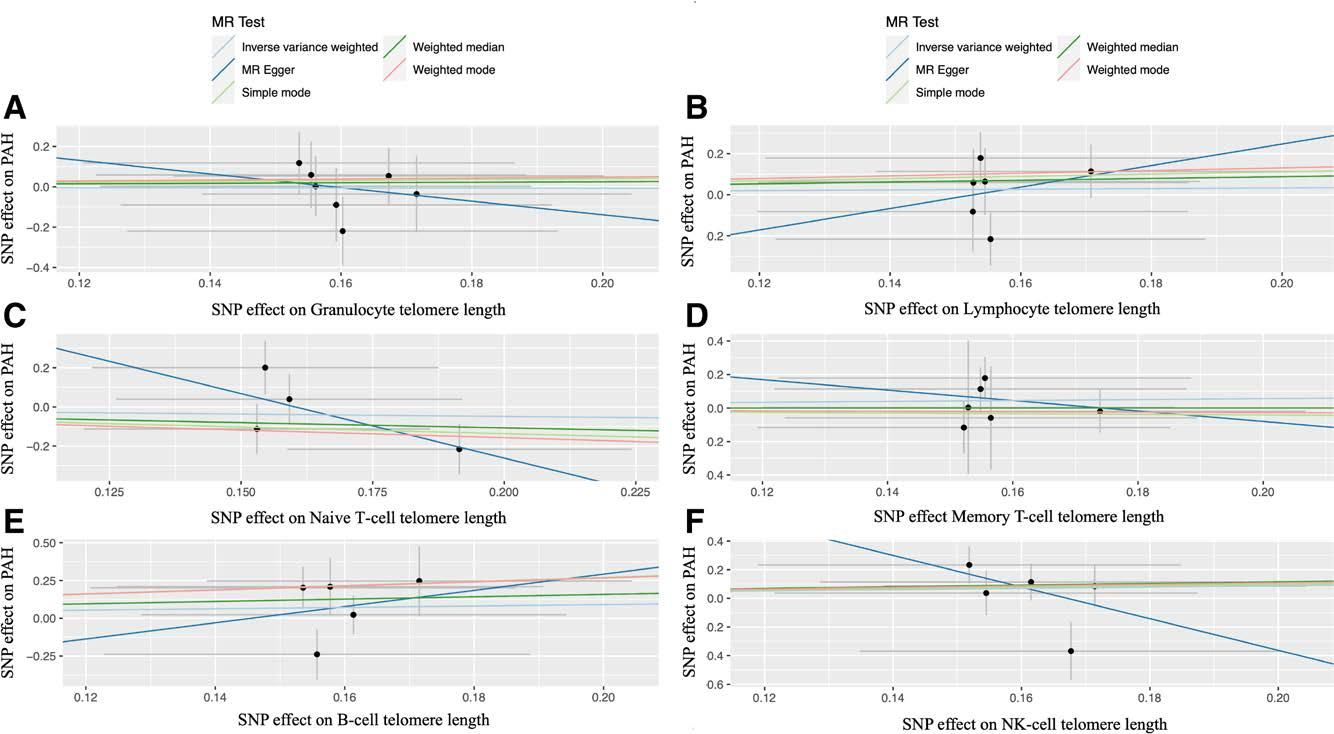
The scatter plot for the causations of other telomere lengths on PAH. *Notes*: The causations of granulocyte telomere length (A), memory T-cell telomere length (B), naive T-cell telomere length (C), lymphocyte telomere length (D), B-cell telomere length (E), NK-cell telomere length (F) on the risk of PAH. MR = Mendelian randomization; NK = natural killer; PAH = pulmonary arterial hypertension; SNP = single nucleotide polymorphism.

The basic information of the included studies is shown in Table 1. In total, 473,076 participants were involved in the present MR analysis (Table 1). As depicted in Table S1 to S7, Supplemental Digital Content, http://links.lww.com/MD/N976 a comprehensive assessment revealed a diverse array of genetic IVs associated with different measures of telomere length. Specifically, the analysis identified 224 IVs linked to overall telomere length (Fig. 2), 5 IVs associated with granulocyte telomere length (Fig. 3A), 7 IVs related to lymphocyte telomere length (Fig. 3B), 6 IVs corresponding to naive T-cell telomere length (Fig. 3C), 4 IVs connected to memory T-cell telomere length (Fig. 3D), 6 IVs for B-cell telomere length (Fig. 3E), and 5 IVs on NK cell telomere length (Fig. 3F). These IVs were presented in detail in the supplementary tables (Tables S1–S7, Supplemental Digital Content, http://links.lww.com/MD/N976). Each of these IVs demonstrated significant associations with their respective telomere length measures, with their F statistics surpassing the threshold of 10 (Tables S1–S7, Supplemental Digital Content, http://links.lww.com/MD/N976). The selected IVs were robust and provided reliable instruments for the MR analysis, thereby enhancing the validity and credibility of the causal inferences drawn between the various telomere length measures and the outcome of interest.

### 3.2. MR analyses for PAH

**Figure 4.**
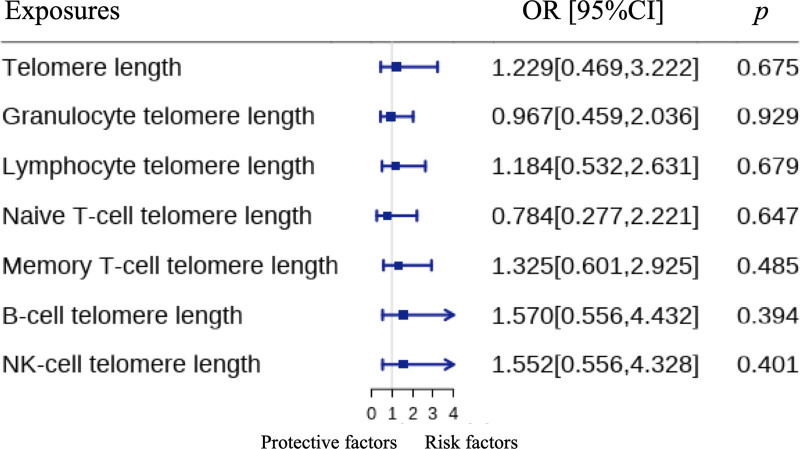
The forest plot for the causations between telomere length and the risk of PAH. CI: confidence intervals; OR: odds ratios; PAH: pulmonary arterial hypertension.

**Figure 5.**
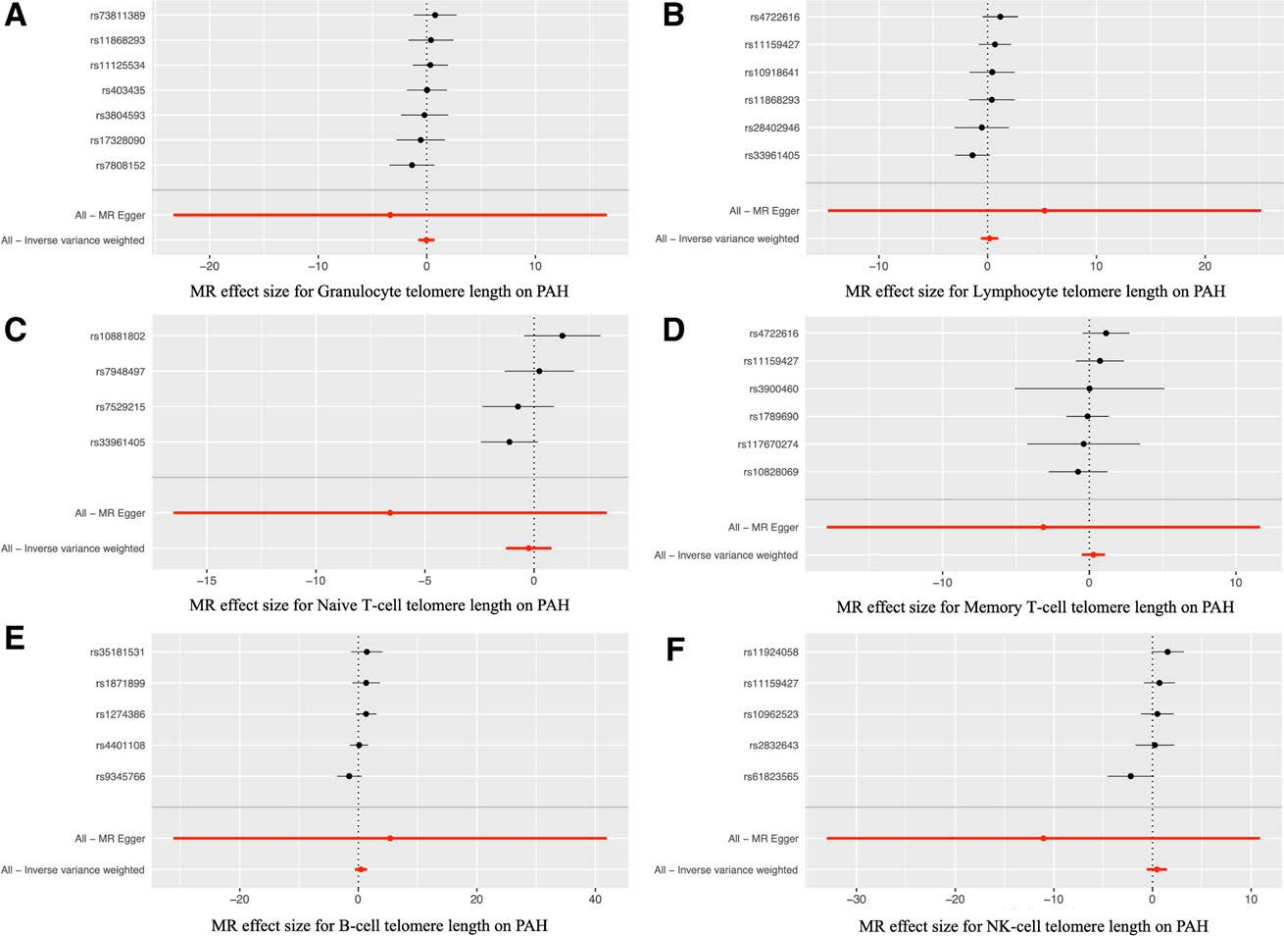
The Mendelian Randomization effect size for the causations between telomere length and PAH. *Notes*: The MR effect size for the causations between granulocyte telomere length (A), memory T-cell telomere length (B), naive T-cell telomere length (C), lymphocyte telomere length (D), B-cell telomere length (E), NK-cell telomere length (F) and the risk of PAH. MR = Mendelian randomization; NK = natural killer; PAH = pulmonary arterial hypertension.

In the MR analyses for PAH, our primary findings from the IVW method revealed no significant causal effects of telomere length on the risk of PAH across various measures (Fig. 4). Specifically, the OR for PAH per SD increase in overall telomere length was 1.229 (95% CI, 0.469–3.222, *P* = .676, Fig. 4 and Fig. S1, Supplemental Digital Content, http://links.lww.com/MD/N977), indicating no significant association. Similarly, granulocyte telomere length (OR = 0.967, 95% CI, 0.459–2.036, *P* = .929, Figs. 4 and 5A), lymphocyte telomere length (OR = 1.184, 95% CI, 0.532–2.631, *P* = .679, Figs. 4 and 5B), naive T-cell telomere length (OR = 0.784, 95% CI, 0.277–2.221, *P* = .647, Figs. 4 and 5C), memory T-cell telomere length (OR = 1.325, 95% CI, 0.601–2.925, *P* = .485, Figs. 4 and 5D), B-cell telomere length (OR = 1.570, 95% CI, 0.556–4.432, *P* = .394, Figs. 4 and 5E), and NK-cell telomere length (OR = 1.552, 95% CI, 0.556–4.328, *P* = .401, Figs. 4 and 5F) all showed no significant causal relationships with PAH. To further validate these results, sensitivity analyses were conducted using the weighted median and MR-Egger methods (Table 2), which confirmed the lack of significant causal associations between the different measures of telomere length and the risk of PAH. Overall, our MR analyses suggest that telomere length does not have a causal effect on the risk of developing PAH.

**Table 2:** The results of sensitivity analysis of the present MR analysis.

Exposure or outcome	Weighted median	MR-Egger	*p*_heterogeneity_*	*p*_Pleiotropy_**
Granulocyte telomere length	1.133[0.464–2.766]	1.554[0.591–4.084]	.748	.756
Lymphocyte telomere length	1.554[0.591–4.084]	0.586[0.211–1.63]	.240	.644
Naive T-cell telomere length	0.586[0.211–1.630]	1.003[0.361–2.783]	.219	.335
Memory T-cell telomere length	1.003[0.361–2.783]	2.213[0.757–6.473]	.615	.675
B-cell telomere length	2.213[0.757–6.473]	1.780[0.640–4.948]	.142	.809
Natural killer -cell telomere length	1.780[0.640–4.948]	2.061[0.479–8.867]	.181	.380

MR = Mendelian randomization.

* The *P*-value for heterogeneity test.

** The *P*-value for pleiotropy.

### 3.3. Effects of individual genetic instruments

**Figure 6.**
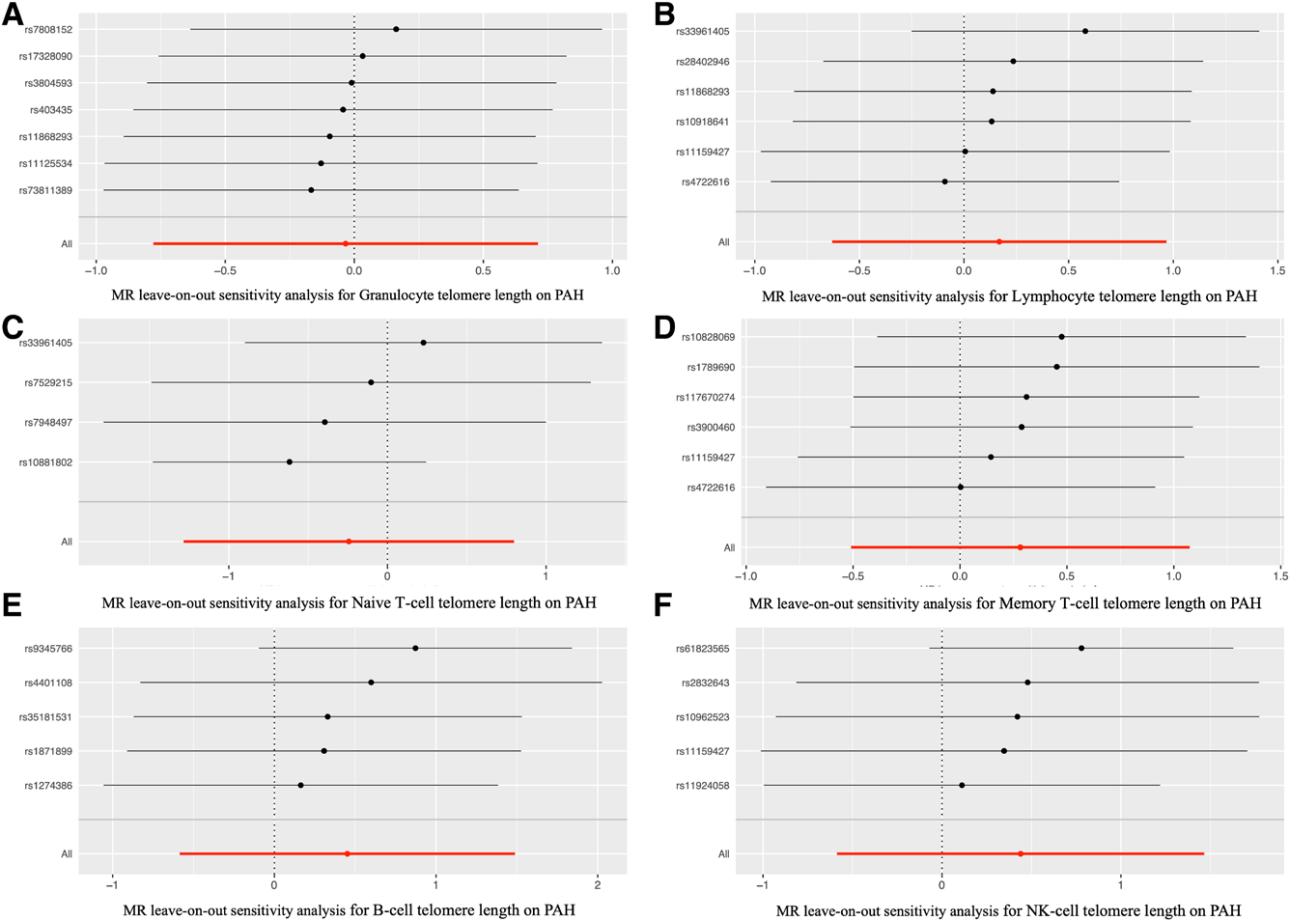
The leave-one-out analysis for the causations between telomere length and PAH. *Notes*: The leave-one-out analysis for the causations between granulocyte telomere length (A), memory T-cell telomere length (B), naive T-cell telomere length (C), lymphocyte telomere length (D), B-cell telomere length (E), NK-cell telomere length (F) and the risk of PAH. MR = Mendelian randomization; NK = natural killer; PAH = pulmonary arterial hypertension.

We further explored the robustness of our findings by conducting a leave-one-out sensitivity analysis (Fig. 6), which involved systematically excluding each independent and significantly associated SNP from our set of genetic instruments and reevaluating the association between telomere length and PAH using the IVW method.

The results of leave-one-out sensitivity analysis demonstrated that the exclusion of any individual SNP did not materially alter the overall estimates. The lack of significant change in the ORs and CIs upon the removal of each SNP underscores the stability and reliability of our findings (Fig. S2, Supplemental Digital Content, http://links.lww.com/MD/N977 and Fig. 6). The consistency observed in the estimates, even after the sequential exclusion of each genetic instrument, reinforces the robustness of our conclusions and supports the assertion that the observed lack of causal association between telomere length and PAH is not driven by any single genetic variant. Leave-one-out sensitivity analysis further strengthens the confidence in the validity of our MR approach and the overall conclusions of the study.

## 4. Discussion

In this study, we conducted a comprehensive MR analysis to investigate the causal association between telomere length and PAH. Our primary analysis utilized the IVW method, alongside sensitivity analyses including the weighted median and MR-Egger regression, to assess the robustness of our findings. Our results consistently demonstrated a lack of significant causations between telomere length and the risk of developing PAH across various measures of telomere length, including overall telomere length, granulocyte telomere length, lymphocyte telomere length, naive T-cell telomere length, memory T-cell telomere length, B-cell telomere length, and NK-cell telomere length. Furthermore, the leave-one-out sensitivity analysis confirmed the stability of these findings, indicating that no single genetic instrument unduly influenced the overall estimates. Collectively, these results provide robust evidence that telomere length does not have a causal effect on the risk of PAH, challenging the notion that telomere biology plays a direct role in the pathogenesis of this condition.

Telomeres, which are protein-DNA complexes located at the ends of linear eukaryotic chromosomes, play a crucial role in maintaining genomic stability and integrity.^[20]^ Comprising repetitive “TTAGGG” sequences, telomeres undergo progressive shortening with each cell division.^[21]^ When telomeres reach a critically short length, cellular consequences such as replicative senescence, genomic instability, and apoptosis are triggered.^[21–23]^ Leukocyte telomere length is commonly used as a surrogate marker for telomere length in various tissues and has been extensively studied as a potential biomarker of human aging.^[24,25]^

Observational studies have suggested associations between leukocyte telomere length and the risk of several age-related diseases, including cardiovascular diseases, type 2 diabetes mellitus, certain cancers, and overall lifespan.^[26–30]^ However, these associations are susceptible to reverse causation and unmeasured confounding due to the nature of observational studies.^[14]^ In the context of PAH, a severe and progressive cardiovascular condition, the relationship between telomere length and disease risk has been of particular interest. While some studies have suggested a potential link between shortened telomeres and increased PAH risk,^[31]^ the causal nature of this association remains uncertain. This study aims to investigate the causal associations between multiple measures of telomere length, including overall telomere length, granulocyte telomere length, lymphocyte telomere length, naive T-cell telomere length, memory T-cell telomere length, B-cell telomere length, and NK-cell telomere length, and the risk of PAH using an MR approach.

The results of this MR study, which starkly contrast with our initial hypothesis, compel us to reconsider the putative association between telomere length and PAH. Initially, we posited that shorter telomeres might contribute to an increased risk of developing PAH, premised on the notion that telomere attrition could mirror broader biological aging processes that negatively impact cardiovascular health. However, the absence of a detectable causal association between telomere length and PAH, despite employing a rigorous methodological framework, underscores the complexity of the genetic landscape underlying PAH.

The findings of our study provide valuable insights into the relationship between telomere length and PAH. Despite previous observational studies suggesting a potential link between shortened telomeres and increased risk of PAH,^[32]^ our MR analysis did not support a causal association. This discrepancy highlights the importance of using methods like MR to distinguish between correlation and causation, as observational studies may be confounded by factors such as drugs, smoking, and other lifestyle variables that can influence both telomere length and PAH risk. The lack of causality observed in our study suggests that while telomere biology may play a role in cellular aging and senescence, its direct impact on the development of PAH may be limited. This finding prompts a reevaluation of the role of telomere length in PAH pathogenesis and suggests that other mechanisms may be more critical in driving the disease process. It also underscores the need for further research to explore the complex interplay between genetic, environmental, and lifestyle factors in the etiology of PAH.

One of the primary advantages of this study is its use of MR analysis, which leverages genetic variants as IVs to infer causal relationships between exposures and outcomes. This approach helps to overcome the limitations of observational studies, such as confounding and reverse causation, providing more robust evidence for causal inference. By using genetic variants associated with telomere length, our study can ascertain whether the observed associations with PAH are likely to be causal. Another strength of this study is the comprehensive selection of telomere length measures, including overall telomere length, granulocyte telomere length, lymphocyte telomere length, naive T-cell telomere length, memory T-cell telomere length, B-cell telomere length, and NK-cell telomere length. This allows for a more nuanced understanding of the relationship between telomere biology and PAH, considering the potential heterogeneity in telomere length dynamics across different cell types. Furthermore, the study benefits from the use of large-scale GWAS data for both the exposure (telomere length) and the outcome (PAH), ensuring a robust and well-powered analysis. The inclusion of sensitivity analyses, such as the weighted median method and MR-Egger regression, adds to the rigor of the study by assessing the robustness of the findings and accounting for potential pleiotropy.

Despite the strengths of this study, several limitations should be acknowledged. First, the use of summary-level data in MR analysis precludes the assessment of potential interactions between genetic variants and environmental or lifestyle factors that might influence the relationship between telomere length and PAH. This limitation restricts our ability to explore the nuanced interplay between genetics and environmental exposures in the context of PAH. Second, while MR provides a powerful tool for causal inference, it relies on several key assumptions, including the absence of pleiotropy and the relevance of the IVs. Although we employed sensitivity analyses to address potential violations of these assumptions, unmeasured pleiotropy or weak instrument bias cannot be completely ruled out. Third, our study focused on a predominantly European population, which may limit the generalizability of the findings to other ethnic or racial groups. Genetic architecture and environmental exposures can vary across populations, potentially influencing the relationship between telomere length and PAH. Fourth, the measures of telomere length used in this study are based on leukocyte telomere length, which may not perfectly reflect telomere dynamics in other tissues relevant to PAH, such as lung or vascular tissues. This limitation could affect the interpretation of the findings in the context of PAH pathogenesis. Lastly, sample size may affect the power of our study, suggesting that larger sample sizes might be required to explore these smaller effects more effectively in future studies.

## 5. Conclusion

Our study employed an MR approach to investigate the causal relationship between telomere length and PAH. The analysis encompassed various measures of telomere length, including overall telomere length and telomere lengths in specific cell types such as granulocytes, lymphocytes, naive T-cells, memory T-cells, B-cells, and NK-cells. Our findings consistently demonstrated a lack of significant causal association between telomere length and the risk of PAH across all measures. These results suggest that telomere biology, as reflected by telomere length, may not play a direct causal role in the development of PAH. This challenges previous assumptions based on observational studies and highlights the importance of using robust methods like MR to clarify the role of biological factors in complex diseases. The study’s findings contribute to a better understanding of the pathogenesis of PAH and suggest that future research should explore other mechanisms, other genetic markers or pathways that may underlie this condition.

## Acknowledgments

We sincerely thank all investigators for sharing the summary statistic data of genome-wide association studies (GWASs).

## Author contributions

**Formal analysis:** Jing-Yang Wang, Jiang-Shan Tan.

**Methodology:** Ting-Ting Lyu.

**Resources:** Ping Qing, Ling-Min Wu, Xiao-Jian Wang.

**Supervision:** Ping Qing, Ling-Min Wu, Xiao-Jian Wang.

**Visualization:** Jing-Yang Wang, Jiang-Shan Tan.

**Writing – original draft:** Ting-Ting Lyu, Jing-Yang Wang, Jiang-Shan Tan.

**Writing – review & editing:** Ting-Ting Lyu, Yan-Min Yang, Yi-Meng Wang, Jing Zhao, Ping Qing, Ling-Min Wu, Xiao-Jian Wang.

## Supplementary Material


