# Supplementary figures and images for: Causal associations between telomere length and pulmonary arterial hypertension: A two-sample Mendelian randomization study
Source: Medicine (Baltimore). 2024 Nov 22;103(47):e40407. doi: 10.1097/MD.0000000000040407 (PMC11596709; doi:10.1097/MD.0000000000040407)

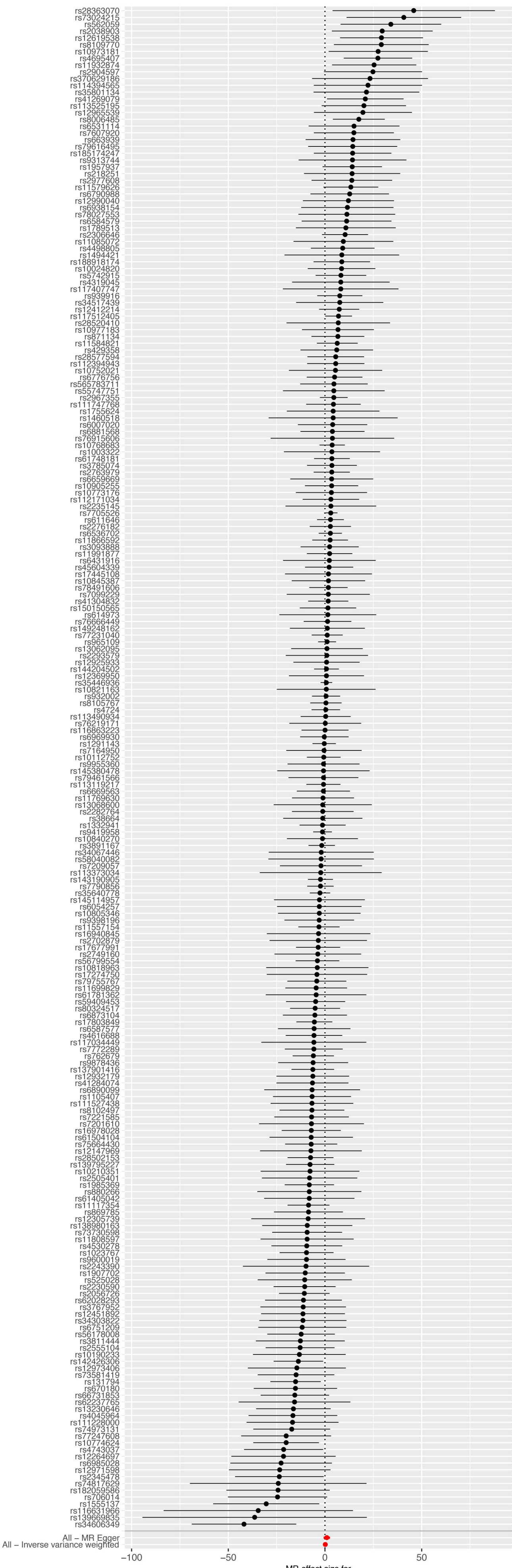

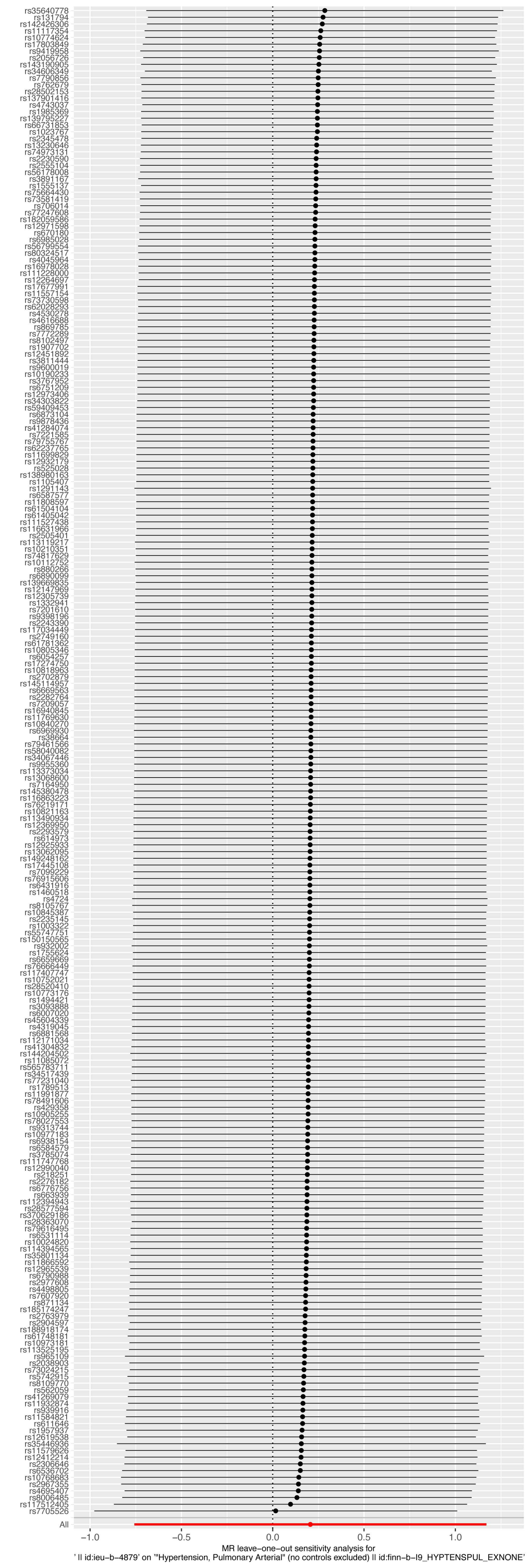

Supplement: Supplementary file 2 [file medi-103-e40407-s002.pdf]
